# Psychopharmacology

**Published:** 2014-08

**Authors:** Shane McInerney, Sidney H Kennedy

**Affiliations:** Toronto, Ontario

**Figure:**
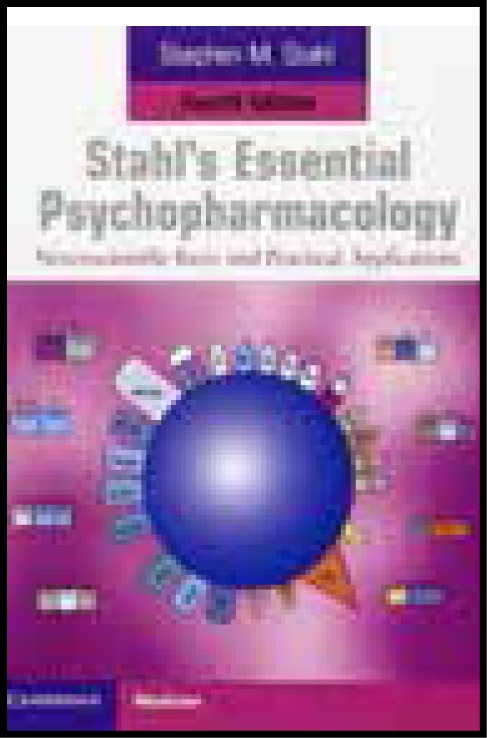


This book comes 5 years after the last edition. It is noticeably slimmer, with a bright attractive cover displaying various receptors waiting to be explored and explained within. While the book is slimmer, the pages are wider, with a new 2-column layout intended to provide greater ease of legibility for the reader.

The previous edition had 8 introductory chapters, with titles such as “Signal Transduction and the Chemically Addressed Nervous System,” with over 200 pages that may have deterred all but the most enthusiastic of readers. In this fourth edition, there are 3 condensed, but no less informative, chapters that address the basics of neurotransmission, receptors, and ion channels as targets of pharmacological drug action. This approach should appeal to the busy clinician who wishes to gain an appreciation of psychopharmacology without feeling overwhelmed by the basic neuroscience of psychopharmacology in standalone chapters. Chapter 1 is highly recommended as a primer for current understanding of the bidirectional signalling cascades between the genome and the neuron. This section also provides an excellent summary of epigenetic and associated molecular mechanisms.

The remaining 11 chapters cover the main domains of psychiatric psychopharmacology, and the author has cleverly supplemented the basic neuroscience of the former introductory chapters into the clinical chapters without overburdening the reader. In keeping with previous editions, the highly recognizable Stahl trademark cartoons of receptor actions appear throughout the text.

The neural circuitry and neurobiology of symptom dimensions in schizophrenia leads nicely into a thorough discussion of current and emerging atypicals. The pharmacological properties of individual antipsychotics (APs) are uniquely described as, “the pines, the dones, two pips and a rip”^p 180^—as an example, clozapine, olanzapine, quetiapine, and asenapine are discussed in the pines chapter, while lurasidone and iloperidone join risperidone, palliperidone, and ziprasidone. Dr Stahl has provided expert guidance on how best to switch from a pine to a done or a pip, and new ideas on using high doses of medication and polypharmacy for treatment-resistant schizophrenia. While the chapter “Antipsychotic Agents” is more than 100 pages long, it is reader-friendly to the clinician who is willing to invest a few hours to become familiar with current rationales, dosing, and side effects for new and emerging APs.

The chapter “Mood Disorders” has expanded detail on the neurochemistry and genetics of the disorder, and the companion “Antidepressants” chapter has been extensively revised to include a new discussion on circadian rhythms and the potential role of melatonin receptors as targets for new antidepressants (ADs), such as agomelatine. Emerging ADs, such as vortioxetine, are introduced, and the relevance of research initiatives using ketamine and deep brain stimulation make interesting reading.

Dr Stahl devotes a section also to mood stabilizers and clarifies that combination therapy for bipolar disorder is often necessary to attain remission. He provides an elegant table with various combinations, ranging from the most evidence-based addition of lithium or valproate to an atypical. Clinical practice combinations, such as li-vo (lithium-valpraote), la-vo (lamotrigine-valproate), as well as geographic-based bipolar combinations, such as the Boston brew (any combination but AD), Californian careful cocktail (any combination with AD), and Tennessee mood shine (atypical plus AD) are discussed. He does point out that lami-quel (lamotrigine plus quetiapine) may be a useful, if somewhat slow-titrating, method of treating bipolar depression.

The “Attention-Deficit Hyperactivity Disorder and Its Treatment” chapter discusses new treatments, such as guanfacine (Intuniv) and lisdexamfetamine (Vyvanse), while the chapter “Dementia and Its Treatment” incorporates both the latest treatments in development and the new diagnostic criteria for Alzheimer disease.

The “Impulsivity, Compulsivity, and Addiction” chapter now includes greater depth on the neurocircuitry of these disorders, along with an update on the available treatments for drug addictions. This chapter exemplifies the book’s new structure into symptom endophenotypes or dimensions of psychopathology that dissect numerous syndromes.

The intergration of the printed word with websites^1^ allows access to downloadable slides of all the figures in the book. In addition, this website is also linked to the *CNS Spectrums* journal, which provides illustrated reviews of current topics in psychiatry, as well as psychopharmacology.

In essence, Dr Stahl has succeeded in updating his textbook on the new developments in the field of psychopharmacology. He continues to be a pioneer in psychopharmacology, and his illustrations and clear explanations of complex neuroscience will continue to attract a wide, international multi-disciplinary readership.
